# Single-Cell and Bulk Transcriptomics Reveal the Immunosenescence Signature for Prognosis and Immunotherapy in Lung Cancer

**DOI:** 10.3390/cancers17010085

**Published:** 2024-12-30

**Authors:** Yakun Zhang, Jiajun Zhou, Yitong Jin, Chenyu Liu, Hanxiao Zhou, Yue Sun, Han Jiang, Jing Gan, Caiyu Zhang, Qianyi Lu, Yetong Chang, Yunpeng Zhang, Xia Li, Shangwei Ning

**Affiliations:** 1 College of Bioinformatics Science and Technology, Harbin Medical University, Harbin 150081, China; 2019020355@hrbmu.edu.cn (Y.Z.); 2022020558@hrbmu.edu.cn (J.Z.); 2022020531@hrbmu.edu.cn (C.L.); zhouhanxiao@hrbmu.edu.cn (H.Z.); 2019020354@hrbmu.edu.cn (Y.S.); 202301212@hrbmu.edu.cn (H.J.); 18245115622@163.com (J.G.); 2020020493@hrbmu.edu.cn (C.Z.); 202401203@hrbmu.edu.cn (Q.L.); 2022020556@hrbmu.edu.cn (Y.C.); zhangyp@hrbmu.edu.cn (Y.Z.); 2 The Second Affiliated Hospital of Harbin Medical University, Harbin 150081, China; jinyitong@hrbmu.edu.cn

**Keywords:** immunosenescence, prognosis, lung cancer, immunotherapy, transcriptomics

## Abstract

Background: Immunosenescence is the aging of the immune system, which is closely related to the development and prognosis of lung cancer. Targeting immunosenescence is considered a promising therapeutic approach. Methods: We defined an immunosenescence gene set (ISGS) and examined it across 33 TCGA tumor types and 29 GTEx normal tissues. We explored the 46,993 single cells of two lung cancer datasets. The immunosenescence risk model (ISRM) was constructed in TCGA LUAD by network analysis, immune infiltration analysis, and lasso regression and validated by survival analysis, cox regression, and nomogram in four lung cancer cohorts. The predictive ability of ISRM for drug response and immunotherapy was detected by the oncopredict algorithm and XGBoost model. Results: We found that senescent lung tissues were significantly enriched in ISGS and revealed the heterogeneity of immunosenescence in pan-cancer. Single-cell and bulk transcriptomics characterized the distinct immune microenvironment between old and young lung cancer. The ISGS network revealed the crucial function modules and transcription factors. Multiplatform analysis revealed specific associations between immunosenescence and the tumor progression of lung cancer. The ISRM consisted of five risk genes (CD40LG, IL7, CX3CR1, TLR3, and TLR2), which improved the prognostic stratification of lung cancer across multiple datasets. The ISRM showed robustness in immunotherapy and anti-tumor therapy. We found that lung cancer patients with a high-risk score showed worse survival and lower expression of immune checkpoints, which were resistant to immunotherapy. Conclusions: Our study performed a comprehensive framework for assessing immunosenescence levels and provided insights into the role of immunosenescence in cancer prognosis and biomarker discovery.

## Simple Summary

Immunosenescence is the decrease in the function of the immune system with age. The immune system plays a key role in the development and progression of tumors. Targeting immunosenescence is considered a promising therapeutic approach to improving tumor prognosis. However, few studies have been conducted to reveal immunosenescence biomarkers of lung cancer. Thus, our study aimed to fill this critical knowledge gap by developing an immunosenescence gene set to characterize tumor immune microenvironment and proposing an immunosenescence risk model to understand the roles of immunosenescence in tumor prognosis and immunotherapy.

## 1. Introduction

Lung cancer remains the leading cause of cancer death worldwide, with a 5-year survival rate of less than 30% [1,2]. The development of immune checkpoint inhibitors (ICIs) has revolutionized lung cancer treatment, but it is difficult to predict patients’ survival risk and response to treatment [3]. The complexity of the immune system and the limitations of existing biomarkers further add to this difficulty [4]. Immunosenescence has attracted great interest as a potential biomarker. However, there is a lack of comprehensive research on immunosenescence in lung cancer. A better understanding of the relationship between immunosenescence and cancer is needed to help identify prognostic and predictive biomarkers and develop more effective treatment strategies for lung cancer patients.

Immunosenescence is the degradation of the innate and adaptive immune systems associated with aging [5]. Immunosenescence has been observed in the tumor microenvironment of mice and humans [6]. Increasing evidence suggests that immunosenescence is associated with cancer progression [7]. It is reported that immunosenescence can accelerate tumor growth by promoting immune escape [8], and can also prevent tumor formation by stimulating immune responses in different environments [9]. Therefore, immunosenescence will improve the targeted therapy and immunotherapy for specific tumors [7,10]. Due to the lack of universal biomarkers of immunosenescence, defining the level of immunosenescence remains a key unanswered question [11,12]. Accurate quantification of the level of immunosenescence in lung cancer is still lacking, especially at the single-cell level [13,14]. In addition, it is still challenging to translate the mechanism of immunosenescence affecting lung cancer into clinical results [15], and frontier research mainly focuses on the process of tumor development. The prediction of the survival risk of patients has become an urgent problem that needs to be solved.

There are prognostic models for predicting lung cancer survival and immunotherapy. For example, deep learning survival neural network models have shown potential advantages in prognostic assessment and treatment recommendations for lung cancer [16]. The model based on glutamine metabolism plays an important role in predicting the prognosis of lung cancer and the efficacy of immunotherapy [17]. The neutrophil prognostic model integrated with single-cell RNA-seq can predict the immune response of non-small cell lung cancer [18]. Targeting immunosenescence is considered a promising therapeutic approach for the prevention of cancer and age-related diseases [19]. However, there is no model based on immunosenescence biomarkers to predict lung cancer prognosis and immunotherapy. Therefore, the identification of clinically important immunosenescence genes will provide potential biomarkers for prognosis prediction and therapeutic targets.

In this study, we defined the immunosenescence gene set (ISGS) and determined the functional annotation ability in multiple tissues. Multi-platform data analysis revealed specific associations between ISGS and tumor progression. The ISRM prediction model can predict survival and immunotherapy treatment across multiple lung cancer cohorts. Our comprehensive analysis provided a valuable framework for better understanding immunosenescence and therapeutic biomarkers.

## 2. Materials and Methods

### 2.1. Data and Resources

Thirty-three TCGA cancer types of RNA-seq data (per million copies of transcript (TPM)) and related clinical information were downloaded from the UCSC Xena browser (http://xena.ucsc.edu/public/, accessed on 10 November 2022) (Supplementary Table S1). The gene expressions of 29 normal tissues were collected in the GTEx (https://www.gtexportal.org/, accessed on 22 November 2022) database (Supplementary Table S2). The bulk transcriptome data were retrieved from the GEO database (GSE68465, GSE72094, GSE26939, GSE68571, GSE40419, and GSE209891) (Supplementary Table S3). For each dataset, we retained samples where both gene expression data and age information were available for subsequent analyses. We retained samples with gene expression and corresponding survival data on predictive performance of the model for prognosis. Thirty-five patients with lung cancers who were treated with the approved PD1-targeting antibodies pembrolizumab or nivolumab were from GSE93157 (Supplementary Table S3). All expression profiles were normalized and log2 transformed. The single-cell RNA-seq datasets of lung cancer were obtained from GSE144945 and the DISCO database (https://www.immunesinglecell.org/, accessed on 5 February 2023) (Supplementary Tables S3 and S4). Three types of immune factors were derived from the TISIDB (http://cis.hku.hk/TISIDB/, accessed on 24 February 2023) database (Supplementary Table S5). The drug sensitivity data of cell lines were obtained from the GDSC (http://www.cancerrxgene.org/, accessed on 13 May 2023) database. We selected 41 non-small cell lung carcinoma (NSCLC) cell lines with age and drug sensitivity data for subsequent studies, of which 10 were sampled at an age of more than 60 and 31 at an age of less than 60 (Supplementary Table S6). The immunohistochemistry (IHC) images of lung cancer were downloaded from the HPA (https://www.proteinatlas.org, accessed on 27 July 2023) database.

### 2.2. Generation of ISGS

The candidate immunosenescence genes were obtained by Genecards (https://www.genecards.org/, accessed on 6 October 2022) and NCBI Gene (https://www.ncbi.nlm.nih.gov/gene/, accessed on 12 October 2022). A manual literature search resulted in a list of 69 studies, from which we identified 90 genes that constitute the ISGS (Supplementary Table S7). The ISGS was generated from genes that were reported to be enriched in immunosenescence cells in previous studies and that were experimentally validated at least in human or mouse cells.

### 2.3. Functional Enrichment Analysis

For each tissue type, samples from TCGA and GTEx were grouped according to the first and fourth quartiles of age. The age groups of GEO cohorts are shown in Supplementary Table S3. Gene set enrichment analysis (GSEA) was performed by “clusterProfiler” (version 4.4.4). Differential gene expression was output using “limma” (v3.52.3) and ordered for each compared gene. The functional gene set used was the ISGS. Finally, the significant enrichment results (adjust. *p* < 0.05 and NES > 1) were visualized by “gseaplot2.” Activity scores of the ISGS were calculated using the “GSVA” package (v1.44.4). Functional enrichment analysis was performed by the Msigdb, Kyoto Encyclopedia of Genes and Genomes (KEGG) pathway, and Gene Ontology (GO) database.

### 2.4. Single-Cell Data Analysis

For the GSE144945 cohort, we used the “FindIntegrationAnchors” and “IntegrateData” functions to integrate the 10 single-cell transcriptome data sets and corrected for batch effects between data sets. After standard quality control, 31,480 high-quality cells were used for clustering analysis. We determined an optimal resolution of 0.5 for clustering with the “clustree” function. The enrichment score of cells was calculated by the R package “irGSEA” (v1.1.3). We used “monocle” (v2.26.0) to build single-cell trajectories. The GeneSwitches method was used to discover the order of gene regulatory events during cell state transitions. For the scRNA-seq data of the DISCO database, after standard quality control and filtration of low-quality cells, a total of 15,513 cells were used for the following analysis. We used the R package “Seurat” (v4.2.0) for data integration and normalization, clustering analysis (resolution = 0.5), and visualization. The “AddModuleScore” function was used to calculate the ISGS score for each cell. “FindAllMarkers” with default parameters were used to identify differentially expressed genes between cells. “CellChat” (v1.5.0) was used for cell–cell communications. Default parameters were chosen to infer the prediction of cell communications.

### 2.5. PPI Network Analysis

In order to identify the crucial ISGS genes at the proteome level, we mapped the 86 protein-coding genes (PCGs) of the ISGS to the protein–protein interaction (PPI) network. The PPI network of the ISGS was constructed by the String (https://cn.string-db.org/, accessed on 2 June 2023) database. The parameter of K-means clustering analysis was set to k = 5. The network analysis was conducted with Cytoscape (v3.9.1). The “enrichplot” (v1.16.2) package was used to identify the significantly enriched biological functions in each cluster. Regulators (motifs and TFs) of the PPI network were predicted with the plugin iRegulon (v1.3) in Cytoscape (v3.9.1). The three databases (TRRUST, RegNetwork, hTFtarget) were integrated to calculate the correlation between TFs and target genes (*p* < 0.05 and r > 0.6).

### 2.6. Construction and Validation of ISRM

LASSO (tenfold cross-validation) was used to select the risk genes for the ISRM. Risk scores of the ISRM were calculated by regression of risk genes: risk score = 0.17689 × (expression of IL7) − 0.33736 × (expression of CD40LG) − 0.14099 × (expression of CX3CR1) + 0.21401 × (expression of TLR3) − 0.07395 × (expression of TLR2). The Kaplan–Meier and log-rank tests were used to evaluate the survival differences. The Cox proportional hazard regression model for overall survival (OS) was constructed with tumor stage, sex, and age as stratification. Survival analysis was performed using the R software packages “survival” (v3.3-1) and “survminer” (v0.4.9). The “pROC” (v1.18.0) and “survivalROC” (v1.0.3) packages were used to evaluate the ROC curves. The “XGBoost” (v1.7.6.1) package was used to predict the accuracy of the ISRM model in response to immunotherapy.

### 2.7. Statistical Analysis

All statistical analyses were performed with the use of R (version 4.2.1).

## 3. Results

### 3.1. Pan-Cancer Analysis Revealed the Heterogeneity Landscape of Immunosenescence

**Figure 1:**
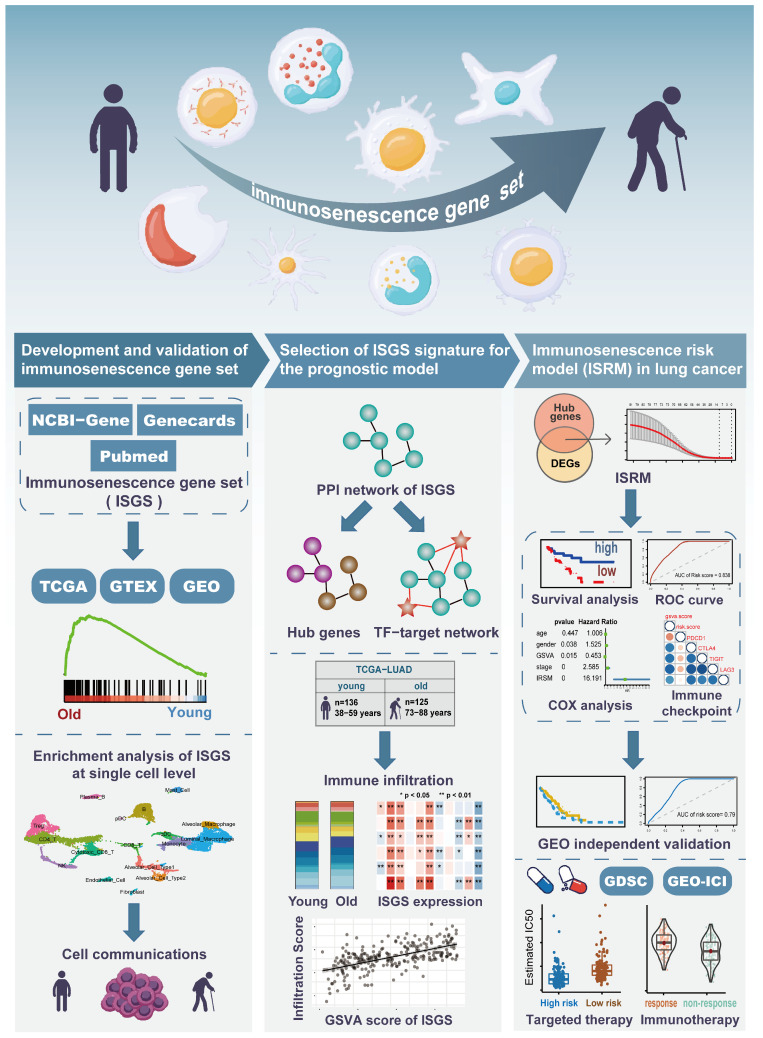
The complete workflow of this study. Our study describes immunosenescence in human transcriptomes, including three sections: function analysis of the immunosenescence gene set in bulk and single-cell transcriptomes, candidate features for immunosenescence prognostic models in lung cancer, and development and validation of the immunosenescence risk model in lung cancer.

**Figure 2:**
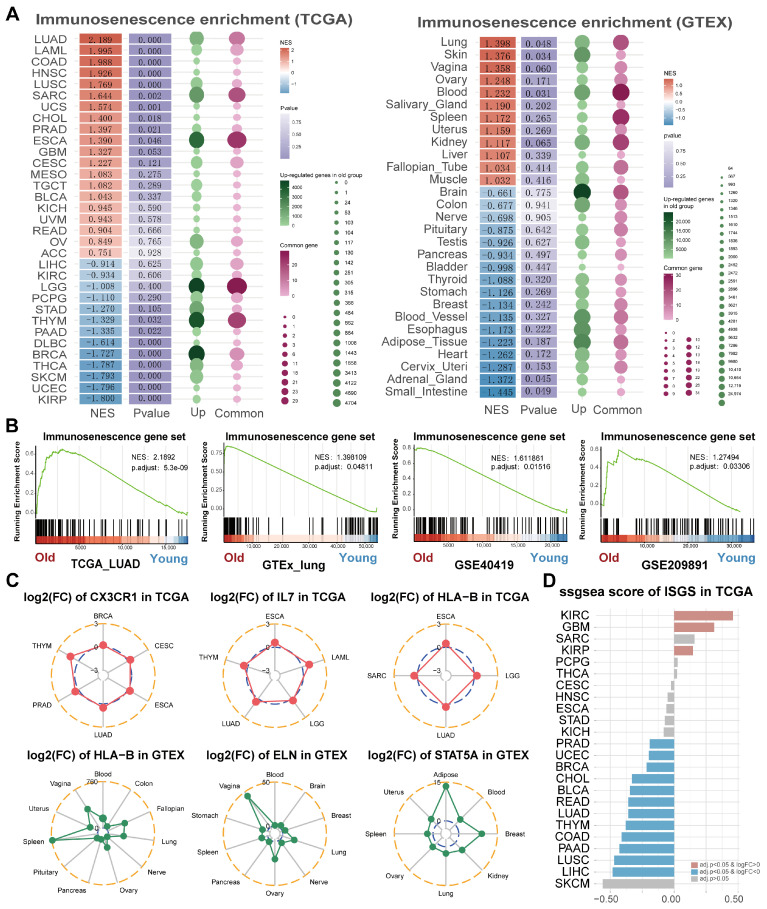
ISGS functional properties associated with aging in TCGA and GTEX. (**A**) The landscape for differential expression and GSEA enrichment scores between age groups in TCGA (left) and GTEX (right). NES represents normalized enrichment score, red means NES > 0, blue means NES < 0; *p* value represents the significance of gene set enrichment; Up represented the number of upregulated genes in old groups; Common represents the number of genes shared by ISGS genes and upregulated genes in the old group. (**B**) Based on GSEA enrichment curves, the ISGS is significantly enriched during the aging process in lung tumors and lung tissues (*p* < 0.05, NES > 1). Red bar means old group; blue bar means young group. (**C**) Radar charts show the log2 (fold change) of ISGS genes in TCGA cancer types (top) and in GTEX normal tissues (bottom). (**D**) Bar plot represents the difference in ssgsea score of the ISGS between TCGA tumors and normal tissues.

The complete workflow of this study is presented in Figure 1. Considering the limitations of single-gene analysis in predicting the complex mechanism of immunosenescence, we generated an immunosenescence gene set (ISGS) to better understand age-related immune dysfunction. Based on a rigorous literature search, a total of 90 genes were developed, consisting mainly of protein-coding genes (PCGs) (*n* = 86) but also miRNA genes (*n* = 4) (Supplementary Table S7). Although these miRNAs may play an important role in immunosenescence [20,21,22], PCGs have a broader range of regulation and tighter interaction (Supplementary Figure S1). We assessed the enrichment applicability of the ISGS in tumor and normal tissues. For the human transcriptome data (TCGA, GTEX, and GSE40419), we defined the old group as the oldest samples in the top 25% and the young group as the youngest samples in the top 25% (Supplementary Tables S1–S3). For the mouse transcriptome data (GSE209891), we defined 3–6 months old as the young group and 12–24 months old as the old group. Notably, both tumor and normal lung tissues were strongly correlated with the ISGS in the old group (*p* < 0.05, NES > 1.3) (Figure 2A). GSEA results confirmed that the ISGS can be used to describe age-related functional properties in lung tissues of different species (*p* < 0.05, NES > 1.0) (Figure 2B). Multiple cancer types in TCGA shared common ISGS genes (CX3CR1, IL7, and HLA-B) (Figure 2C). HLA-B, ELN, and STAT5A were also significantly upregulated in multiple GTEX tissue types. We calculated ISGS scores for TCGA tumors and normal tissues by the ssgsea method, and variance analysis indicated that immunosenescence had inter-tumor heterogeneity in pan-cancer (Figure 2D). These results suggest that ISGS genes may be strongly associated with immunosenescence in lung cancer.

### 3.2. ISGS Defined the Cell Clusters with Immunosenescence Characteristics at High Resolution

**Figure 3:**
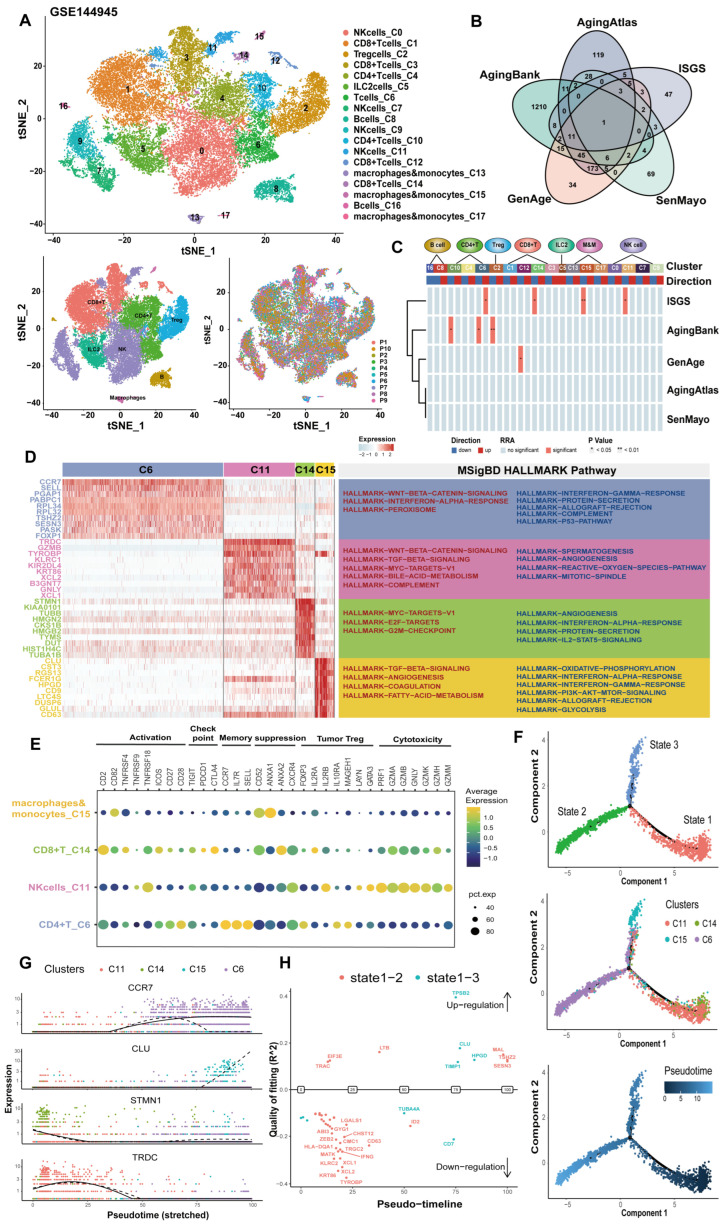
Four ISGS-related cell clusters were identified in single-cell data of lung cancer. (**A**) The tSNE plot of single-cell clustering analysis of GSE144945. (**B**) The number of overlapping genes of senescence-related gene sets. (**C**) To assess whether gene sets were enriched in cell subsets, we scored individual cells using four gene set enrichment methods and then calculated the differentially expressed gene sets for each cell subset. Finally, we used the robust rank aggregation (RRA) algorithm to screen out the gene sets that were significantly enriched in most gene set enrichment analysis methods. (**D**) The top 10 expression (left) and enriched (right) pathways of the markers of immunosenescence clusters. Red Hallmark represents the upregulated pathway, blue Hallmark represents the downregulated pathway. (**E**) The dot plot shows the average expression of cell molecules in the immunosenescence clusters. (**F**) The pseudotime trajectory of immunosenescence clusters annotated by states (top), clusters (middle), and pseudotime (bottom). (**G**) The expression of the top 1 marker of immunosenesence clusters during the pseudotime. (**H**) The different regulation of switch genes between two branches (state1–2 and state1–3).

Single-cell sequencing can be used to study individual cells, thereby revealing the biological processes within cells and intra-tumor heterogeneity. We integrated 31,480 single cells from 10 lung cancer patients in GSE144945 and clustered them into 18 clusters after batch correction (Figure 3A). We manually annotated seven immune cell types (Supplementary Figure S2), and these cell clusters differed in number and function (Supplementary Figure S3A–C). We compared four senescence-related gene sets (AgingAtlas [23], AgingBank [24], GenAge [25], and SenMayo [26]) to validate the efficacy of the ISGS, and there was only one overlapping gene between the five gene sets (Figure 3B). We used the irGSEA method to calculate enrichment scores (Supplementary Figures S3D and S4A), and four clusters were significantly enriched in the ISGS, with C6 being upregulated, while C11, C14, and C15 were downregulated (Figure 3C). Next, we performed the functional analysis for the markers of immunosenescence clusters (Figure 3D). These clusters expressed different immune molecules: high expression of memory molecules in CD4+ T_C6, cytotoxic factors in NK_C11, immune checkpoints in CD8+ T_C14, and suppressive factors in Macrophages&monocytes_C15 (Figure 3E and Supplementary Figure S4B). The trajectory analysis of immunosenescence clusters exhibited three distinct differentiation processes (Figure 3F). The expression of markers changed with pseudotime (Figure 3G). We identified and compared the expression of switch genes that regulate developmental differentiation in two branches (state1–2 and state1–3) using the Geneswitches approach (Figure 3H and Supplementary Figure S4C). These results indicate that the immunosenescence microenvironment enriched a large number of CD4+ memory T cells but reduced the function of CD8+ T cells and NK cells.

### 3.3. ISGS Uncovered Distinct Immunosenescence Microenvironment Accosiated with Aging

**Figure 4:**
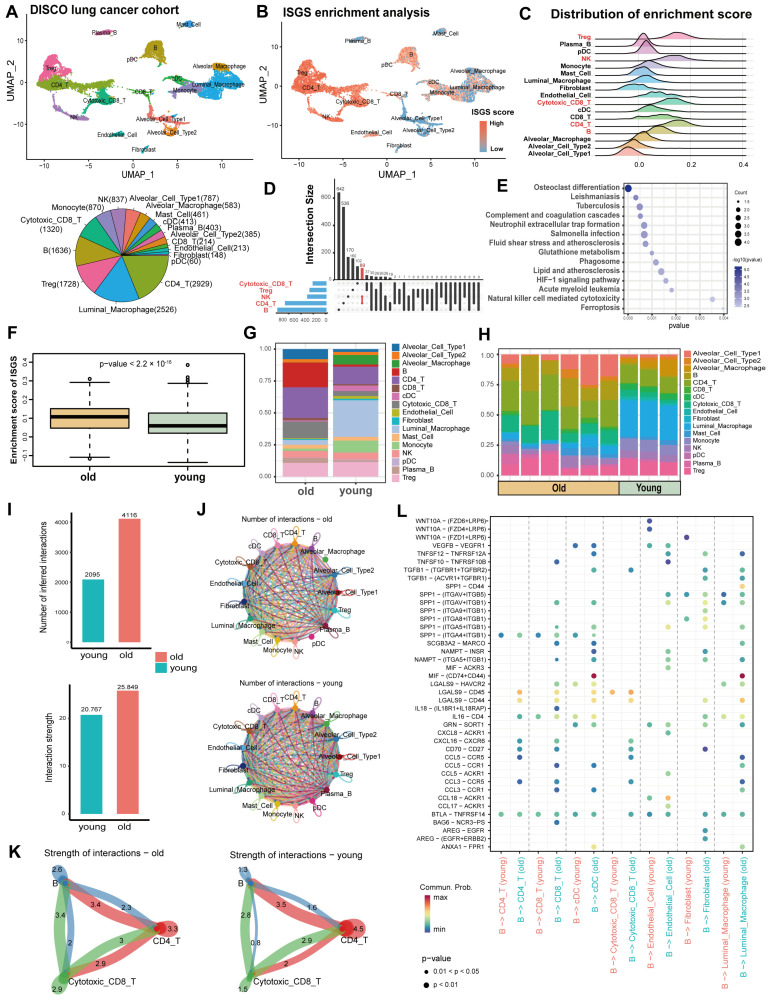
Immunosenescence-associated characteristics in tumor immune microenvironments. (**A**) Uniform manifold approximation and projection (UMAP) plot showing the main cell types in single-cell datasets of lung cancer patients (top). Proportions of cell clusters, with the numbers in parentheses indicating the number of cells (bottom). (**B**) The enrichment score (ES) for the ISGS within cell clusters, represented in the UMAP plot. (**C**) The distribution of ISGS enrichment scores of cell clusters. (**D**) The number of shared marker genes among the top 5 ISGS enriched cells. (**E**) KEGG pathways that were significantly enriched by the markers of the top 5 ISGS enriched cells. (**F**) Box plot of ES between the old and young groups. (**G**) The proportions of cell sub-populations between age groups. (**H**) The proportions of cell sub-populations among samples. (**I**) The inferred interaction number (top) and strength (bottom) between old and young groups. (**J**) Inferred cell–cell interactions among cell clusters in groups. (**K**) The crosstalk of the tumor-infiltrating lymphocyte cells (cytotoxic CD8 + T cells, CD4+ T cells, and B cells). The numbers represent the relative interaction strength as the sum of interaction weights. Edge weights are proportional to interaction strength; a thicker line refers to a stronger signal. (**L**) Dot plot for LRIs between B cells and other cells comparing old and young groups.

The subtle difference in immunosenescence was characterized by scRNA-seq from nine lung tumors (Supplementary Table S4). After standard processing and quality filtering of the raw sequencing data, 15,513 cells were retained for analysis. Unsupervised graph-based clustering and visualization revealed 17 distinct cell clusters (Figure 4A and Supplementary Table S8). The ISGS enrichment score for each cell was calculated to evaluate the immunosenescence level (Figure 4B). The top five cell clusters were Treg (Regulatory T cell), CD4_T, NK (natural killer), cytotoxic_CD8_T, and B cells (Figure 4C). These clusters have a higher degree of immunosenescence and may express more immunosenescence markers (Figure 4D,E). The number and function of immune cells will change with aging [7]. We compared immune cells and ISGS scores between age groups. The overall level of immunosenescence in the old group was significantly higher than that in the young group (*p* < 2.2 × 10^−16^) (Figure 4F). The percentage of cell abundance also showed heterogeneity between groups (Figure 4G) and individuals (Figure 4H). Tumor-infiltrating lymphocytes (TILs) were more abundant in the old group and the immunosuppressive function of TILs with a tumor-killing effect and the secretion of cytokines were both reduced. Therefore, targeting TILs can improve the anti-tumor immune response in lung cancer patients [27,28,29]. We found cell interactions were generally increased and stronger in the old group (Figure 4I,J). Potential ligand–receptor interactions (LRIs) between TILs were inferred with CellChat (Figure 4K). LRI signals associated with B cells were examined, and the interactions of SPP1 were abundant in the old group and may promote immunosenescence (Figure 4L). SPP1 is closely related to B cell infiltration and the expression of immunosuppressive cytokines [30]. In addition, frequent upregulation of SPP1 in cancer leads to poor prognosis [31] and may be suitable as a therapeutic target for chemoradiotherapy resistance [32].

### 3.4. ISGS Was Closely Related to Tumor Immune Infiltration in Lung Cancer

**Figure 5:**
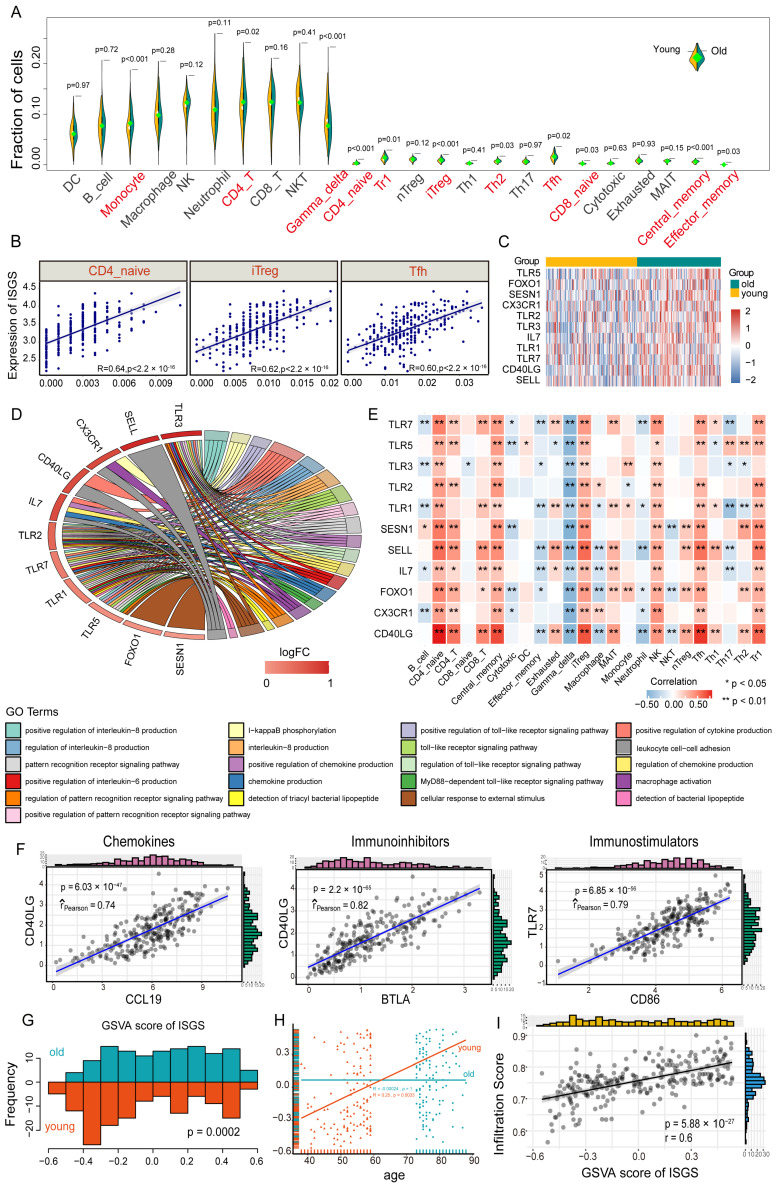
ISGS activity was significantly correlated with the immune infiltration in lung adenocarcinoma. (**A**) Fraction of immune cell infiltration between old and young samples in the TCGA LUAD cohort. Green represents the old group; yellow represents the young group. (**B**) The overall activity of the ISGS is positively correlated with immune cells (*p* < 0.05, R > 6.0). The scatter represents the correlation coefficient. (**C**) The heat map shows that the expression of ISGS genes was significantly upregulated in old samples. Green represents the old group; yellow represents the young group. (**D**) Functional annotation of upregulated ISGS genes. GO terms show the biological process (BP). Red bar means log2 (fold change). (**E**) Correlation between upregulated ISGS genes and immune cell infiltration. (**F**) Scatter plots between ISGS genes and immune factors. (**G**) GSVA scores for the ISGS differed significantly between old and young groups (*p* < 0.05, Wilcoxon rank sum test). (**H**) Scatter plots of GSVA scores in age groups. (**I**) Pearson correlation between the immune infiltration score and the GSVA score of the ISGS. GSVA, gene set variation analysis.

To explore the association of the ISGS and tumor immune microenvironment (TME) in lung cancer, TCGA LUAD was used to estimate the abundance of 24 immune cells (Figure 5A and Supplementary Figure S5A). The abundance of 11 immune cells was significantly different between the age groups (*p* < 0.05) (Figure 5A). The abundance of CD4+ T cell subsets (CD4_naive, iTreg, and Tfh) and ISGS expression showed a significant positive correlation (r > 0.6, *p* < 0.05) (Figure 5B). We speculated that higher immunosenescence levels are associated with more tumor-infiltrating CD4 naive T cells and more differentiation of iTreg and Tfh cells. We identified 11 significantly upregulated ISGS genes in the old group (*p* < 0.05) (Figure 5C, Supplementary Figure S5B). These genes may regulate important biological processes such as cytokine production and toll-like receptor signaling pathways (Figure 5D). Correlation analysis showed that up-regulated genes were negatively correlated with Gamma_delta cells and positively correlated with CD4_naive, Central_memory, iTreg, and NK cells (*p* < 0.05) (Figure 5E). Gamma_delta T cells are small in number but powerful in function, especially in tumor immunity [33]. In addition, high expression of CD40LG was significantly positively correlated with the infiltration of multiple regulatory CD4+ T cell subsets (*p* < 0.01) (Figure 5E). CD40LG might be a powerful predictive biomarker of tumor immunosenescence.

We assessed the expression correlation between upregulated ISGS genes and immune factors (chemokines, *n* = 41; immunostimulator, *n* = 45; and immunoinhibitor, *n* = 24; for more details, see Supplementary Table S5) (Supplementary Figure S5C–E). Gene pairs with the strongest positive correlation were identified: CD40LG-CCL19 (r = 0.74, *p* < 0.05), CD40LG-BTLA (r = 0.82, *p* < 0.05), and TLR7-CD86 (r = 0.79, *p* < 0.05) (Figure 5F). We used the gene set variation analysis (GSVA) algorithm to assess the activity of the ISGS in individual samples separately, and the GSVA scores showed a significant difference between age groups (*p* = 0.0002) (Figure 5G). Moreover, the GSVA scores increased with age in the young group, but there was nearly no change in the old group (Figure 5H). Moreover, the GSVA scores showed a significant positive correlation with the immune infiltration scores (r = 0.6, *p* = 5.88 × 10^-27^) (Figure 5I). Therefore, the activity of the ISGS was closely associated with aging TME, and tumors with a higher degree of immunosenescence may infiltrate more senescence immune cells.

### 3.5. ISGS Showed Consistent Function in the Protein–Protein Interaction and Transcriptional Regulatory Network

**Figure 6:**
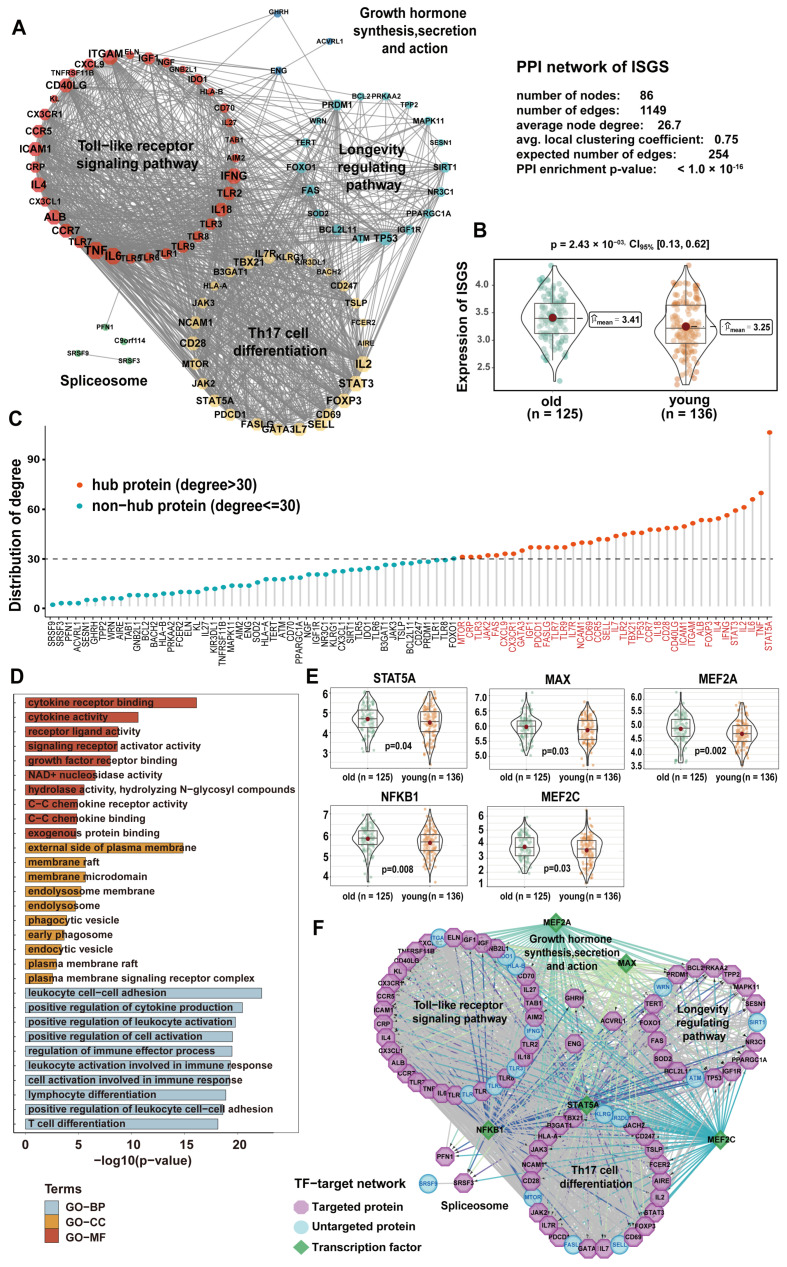
Protein-protein interaction (PPI) network and TF-target network associated with the ISGS. (**A**) The PPI network consists of ISGS genes via string analysis. (**B**) Overall expression levels of ISGS genes between young and old groups (*t*-test, *p* < 0.05). (**C**) The lollipop chart shows the degrees of nodes in the PPI network. (**D**) Hub genes significantly enriched in GO terms (BP, biological process; CC, cellular component; MF, molecular function). (**E**) Boxplots of the expressions of TFs between old and young groups. (**F**) The TF-target network consists of ISGS genes and TFs.

To investigate the overall role of the ISGS in transcriptional regulation and gene expression, we constructed an immunosenescence-related protein–protein interaction (PPI) network and a transcriptional regulation network. The PPI network was generated by 86 PCGs of the ISGS based on the STRING database (Figure 6A). Clustering and functional analysis showed that the PPI network was composed of five clusters with different biological functions (Supplementary Figure S6). We evaluated the overall ISGS gene expression by TCGA LUAD (*n* = 261). Compared with young samples (*n* = 125), the ISGS was significantly highly expressed in old samples (*n* = 136) (*p* = 2.43 × 10^−3^, *t*-test) (Figure 6B). The nodes whose degree exceeded 30 were defined as hubs (*n* = 37), and STAT5A (degree = 109) was identified as the most critical hub gene in the network (Figure 6C). STAT5A is an immunosenescence gene that performs the function of signal transduction and transcription activation and plays an important role in adaptive immunity [34]. GO enrichment results exhibited that hub genes mainly regulated immune activation processes and receptor-binding signals (Figure 6D). Next, key regulatory elements and motifs of the ISGS were identified by iRegulon (Supplementary Table S9). Five main transcription factors (TFs) were significantly upregulated in the old group (*p* < 0.05, *t*-test) (Figure 6E). STAT5A, as the most connected gene in the PPI network, was also the key TF of the ISGS. The TF-target network of the ISGS showed the leading regulation for a majority of the ISGS (Figure 6F). We speculated that these TFs positively regulated ISGS signals by activating the transcription of immunosenescence genes (Supplementary Table S10).

### 3.6. Construction and Validation of ISRM in Multiple Lung Cancer Cohorts

**Figure 7:**
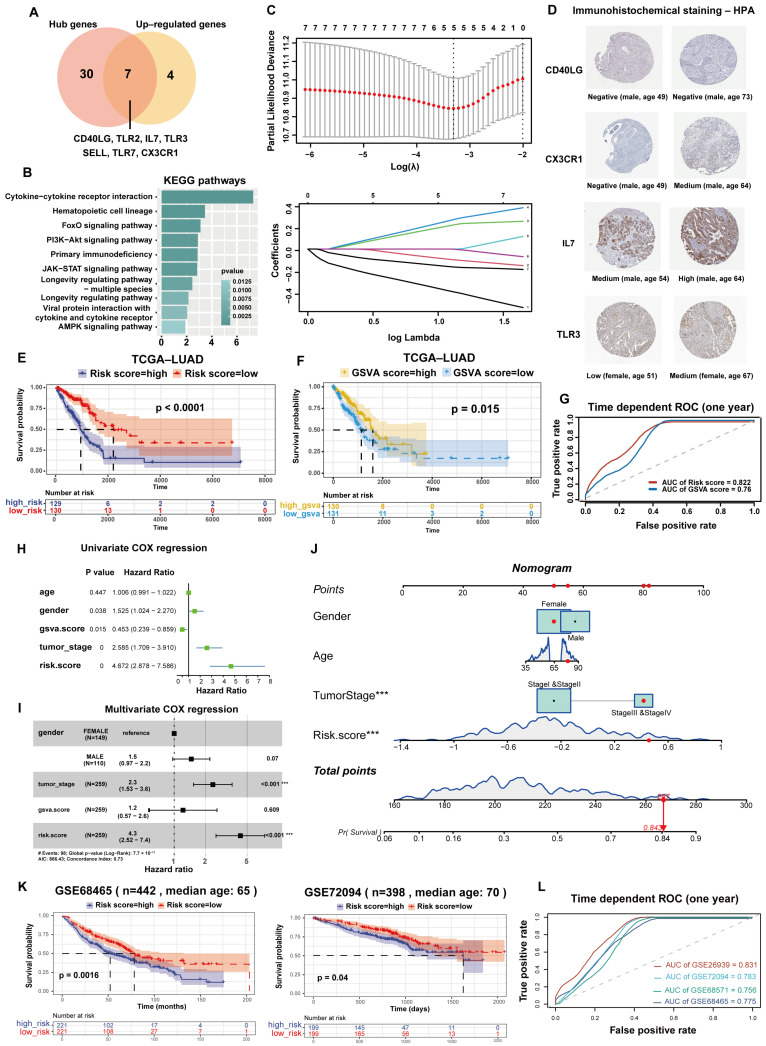
Construction and validation of the immunosenescence risk model (ISRM) in lung cancer cohorts. (**A**) Key ISGS genes were selected as candidate features for the model. (**B**) KEGG pathways are enriched by feature genes, showing the top ten pathways. (**C**) LASSO regression analysis identified 5 risk genes for the ISRM. (**D**) IHC staining of risk genes (CD40LG, CX3CR1, IL7, TLR3) in LUAD. (**E**) Kaplan–Meier plots of overall survival grouped by the median of the risk scores. Blue represents the high-risk group; red represents the low-risk group. (**F**) Kaplan–Meier plots of overall survival grouped by the median of the GSVA scores. Yellow represents the high-score group; light blue represents the low-score group. (**G**) ROC curves for one-year survival rate in TCGA LUAD patients. Red means the IRSM prediction model, blue means the GSVA prediction model. (**H**) Univariate Cox regression analysis for the ISRM and clinical factors. (**I**) Multivariate Cox regression analysis for the ISRM and clinical factors. ***: *p* < 0.001. (**J**) A constructed nomogram for prognostic prediction of a patient with LUAD. The importance of each variable was ranked according to the standard deviation along nomogram scales. ***: *p* < 0.001. (**K**) Kaplan–Meier curves for overall survival grouped by the risk scores in GSE68465 and GSE72094 (*p* < 0.5, log-rank test). (**L**) ROC curves for one-year survival rate in GSE68465, GSE72094, GSE26939, and GSE68571. TCGA, The Cancer Genome Atlas; ROC, receiver operating characteristic; LUAD, lung adenocarcinoma; AUC, the area under the ROC curve.

To facilitate the clinical application of the ISGS, seven upregulated hub genes from the ISGS were selected as candidate features for prognostic models (Figure 7A). The candidate features were mainly enriched in cytokine interaction, classical oncogenic signaling pathways, and longevity-regulating pathways (Figure 7B). The TCGA LUAD cohort was used as the training set, and 1041 patients from four lung cancer cohorts (GSE68465, GSE72094, GSE26939, and GSE68571) were used as independent validation sets.

We defined the ISRM as follows: risk score = 0.17689 × (expression of IL7) − 0.33736 × (expression of CD40LG) − 0.14099 × (expression of CX3CR1) + 0.21401 × (expression of TLR3) − 0.07395 × (expression of TLR2) (Figure 7C). Immunohistochemistry (IHC) staining from The Human Protein Atlas (HPA) database demonstrated that four risk genes were highly expressed in old samples with lung cancer (older than 60 years) (Figure 7D). Patients were then divided into high-risk and low-risk groups according to the median risk score. In the TCGA training set, patients with higher risk scores had significantly worse survival and faster disease progression (*p* < 0.05, log-rank test) (Figure 7E). To assess the impact of immunosenescence activity on overall survival, patients were divided into high-score and low-score groups based on the median GSVA score. In contrast to the risk score, patients with high GSVA scores had better survival (*p* = 0.015, log-rank test) (Figure 7F). The one-year receiver operating characteristic (ROC) curve of the risk score (AUC = 0.822) was higher than the GSVA score (AUC = 0.76), suggesting that the predictive power of the ISRM was better than that of the GSVA score (Figure 7G).

To further validate the prognostic ability of the ISRM, clinical characteristics (age, gender, and tumor stage) of patients were included in univariate Cox regression analysis. The risk score was significantly associated with a worse prognosis and demonstrated clinical independence (*p* < 0.05, HR = 4.672, 95% CI = 2.878–7.586) (Figure 7H). The multivariate Cox proportional hazard model showed that the risk score was the most significant risk factor (*p* < 0.001, HR = 4.3, 95%, CI = 2.52–7.4) (Figure 7I). The nomogram indicated that the ISRM may have promising clinical applications in prognostic prediction (Figure 7J). The classification ability of ISRM was further examined by principal component analysis (PCA) (Supplementary Figure S7A). The risk scores and immune checkpoint molecules showed inverse correlations (Supplementary Figure S7B), which implied that high-risk groups were less likely to benefit from immunotherapy. For each validation dataset, patients with high-risk scores had a significantly higher proportion of deaths than samples with low-risk scores, which was consistent with the training set (Figure 7K, Supplementary Figure S7C,D). The overall survival of GSE68465 and GSE72094 were significantly different between high-risk and low-risk groups (*p* < 0.05). The one-year AUCs of four validation datasets were all above 0.75 (Figure 7L). These results indicate that the predictive power of the ISRM for overall survival showed stability and robustness. The ISRM can play a stable role in predicting prognosis across multiple lung cancer cohorts.

### 3.7. ISRM Improved Potential Anti-Tumor Therapy and Immunotherapy of Lung Cancer

**Figure 8:**
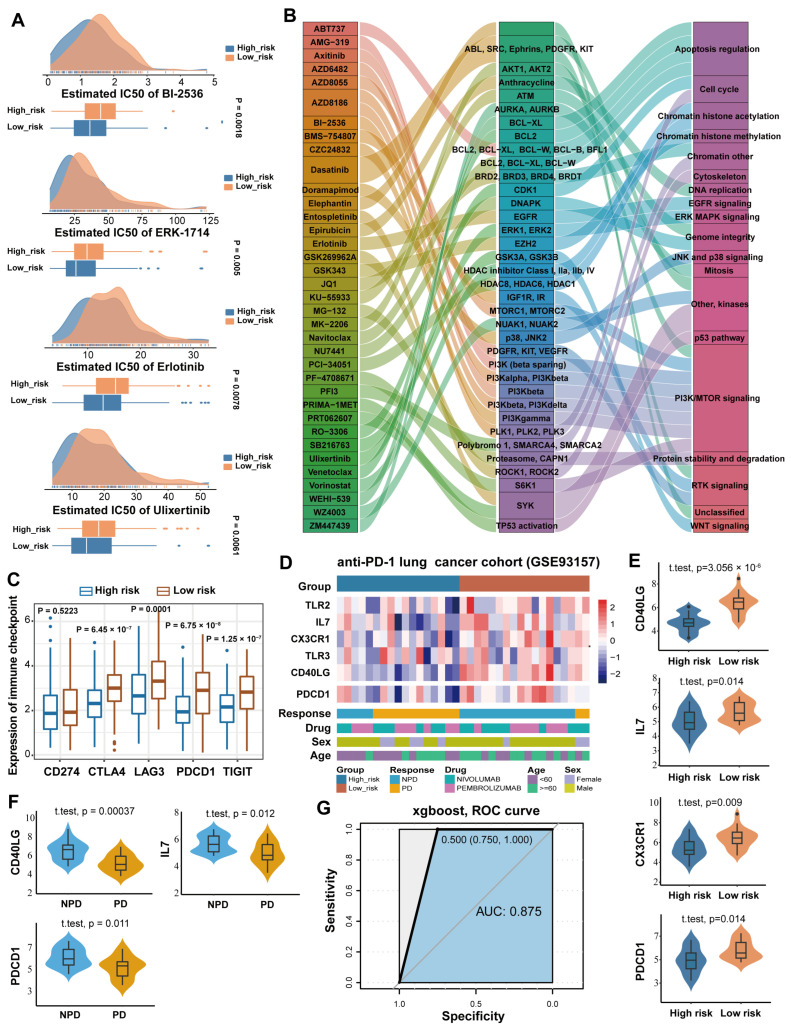
Application of the ISRM in anti-tumor therapy and immunotherapy of lung cancer. (**A**) Density plots and boxplots of high-risk group-specific anti-tumor drugs predicted by the ISRM model. Blue represents the high-risk group; orange represents the low-risk group. (**B**) Anti-tumor drugs (left) with significant IC50 differences between risk groups, targets (middle), and pathways (right). (**C**) Boxplots of immune checkpoint molecules grouped by risk scores. Blue represents the high-risk group, orange represents the low-risk group. (**D**) Heatmap showing the expression of the risk genes and PDCD1 in the GSE93157 cohort of 35 patients. Blue represents the high-risk group; orange represents the low-risk group. (**E**) Violin plots of risk genes and PDCD1 grouped by the ISRM prediction model. Blue represents the high-risk group; orange represents the low-risk group. (**F**) Violin plots of risk genes and PDCD1 grouped by the response to anti-PD-1 immunotherapy. Blue means responder (NPD), and yellow means non-responder (PD). (**G**) XGBoost evaluated the predictive ability of ISRM on immunotherapy. IC50, the half maximal inhibitory concentration.

Accumulating evidence suggests that immunosenescence is associated with resistance to immunotherapy and anti-tumor drug therapy [3,8,35]. Drug sensitivity analysis of 173 anti-cancer drugs by the oncoPredict algorithm showed that the response of high-risk and low-risk groups to 39 anti-cancer drugs was found to be significantly different (*p* < 0.01, *t*-test) (Supplementary Figure S8). Patients in the high-risk group were more likely to benefit from Ulixertinib, BI-2536, ERK-1714, and erlotinib (Figure 8A). We used the Genomics of Drug Sensitivity in Cancer (GDSC) database to predict the potential targets and signaling pathways of anti-cancer drugs that differed between risk groups (Figure 8B). For example, BI-2536 was used in the treatment of various cancers by perturbing the cell cycle through PLK1/2/3. We compared the expression of immune checkpoints between the high-risk and low-risk groups in TCGA LUAD. The expressions of CTLA4, LAG3, PDCD1, and TIGIT in the low-risk group were significantly higher than those in the high-risk group (*p* < 0.05) (Figure 8C), suggesting that the low-risk group was more sensitive to immune checkpoint inhibitors. Forty-one NSCLC cell lines from the GDSC database were used to analyze the response of cell lines to drugs. We divided the cell lines into old and young groups according to whether they were over 60 years old or not, and the IC50 of 20 drugs showed a significant difference between age groups (*p* < 0.05) (Supplementary Figure S9). These findings suggested that the ISRM showed good performance in precision medicine of lung cancer.

Immune checkpoint inhibitors (ICIs), particularly those targeting programmed death 1 (PD-1), produce durable responses and enhance anti-tumor effects in a large number of patients with non-small-cell lung cancer. We collected anti-PD-1 therapy data from 35 lung cancer patients, of which 18 patients were treated with nivolumab and 17 patients were treated with pembrolizumab. The patients were divided into high-risk and low-risk groups according to the ISRM. We found a strong correlation between the ISRM low-risk group and the ICI-benefited group (NPD, nonprogressive disease), as well as between the ISRM high-risk group and the ICI no-benefit group (PD, progressive disease) (Figure 8D). Risk genes (CD40LG, IL7, and CX3CR1) and PDCD1 were highly expressed in the low-risk group (*p* < 0.05) (Figure 8E). The expression of CD40LG, IL7, and PDCD1 were significantly higher in the ICI treatment response group (*p* < 0.05) (Figure 8F). The XGBoost algorithm was used to construct the prediction model of ICI treatment response. The five-fold cross-validation method was used to evaluate the performance of the model, and the AUC value was 0.875 (Figure 8G). In conclusion, the ISRM could be used to predict the treatment outcome of lung cancer by anti-cancer drug therapy and immunotherapy in the future. The ISRM provides new insights into prognostic assessment in lung cancer.

## 4. Discussion

Immunosenescence is an important biological process in cancer and immunity [36]. Characterization of immunosenescence has been problematic for several reasons, especially in bulk RNA-seq [11,37] or scRNA-seq data [6]. There is a lack of a consistent list of genes to reliably identify immunosenescence. To gain insight, we generated an ISGS and validated it in multiple cohorts (TCGA, GEO, and GTEX). We demonstrated the applicability of the ISGS across tissues and species. Next, we applied the ISGS to the lung cancer scRNA-seq data. We characterized immune cells that highly express immunosenescence markers and analyzed the communication pattern between immunosenescence cells. The ISGS activity and interaction strengths of TILs were generally higher in the old group. Moreover, upregulation of SPP1 on B cells in cancer is associated with poor prognosis and may be a potential target for anti-tumor therapy [38]. STAT5A, as the most connected hub gene in the ISGS network, is also the main TF that regulates the ISGS. STAT5A regulated immunosenescence signaling by affecting adaptive immune processes [34]. Therefore, STAT5A is an important regulator of immunosenescence and can be used as a potential target for treatment in clinical practice. We found that the abundance of adaptive immune cells in tumors was higher in older patients [39]. In addition, the upregulation of ISGS genes was accompanied by a decrease in Gamma_delta T cells and an increase in CD4_naive, Central_memory, iTreg, and NK cells. In particular, high expression of CD40LG was significantly associated with an increased number of CD4+ T cell subsets and with the expression of immune checkpoint molecules [40]. Thus, CD40LG is also a powerful predictive biomarker of immunosenescence.

The specific association of immunosenescence with cancer could guide the rational selection of targeted therapies for specific cancer types [41]. To clarify the association between the ISGS and clinical outcomes of cancer, we constructed the ISRM to predict patient survival and treatment response. The ISRM improved the survival stratification of patients in different lung cancer cohorts (TCGA training set and four GEO testing sets), suggesting potential prognostic biomarkers. Patients in the high-risk group had worse survival outcomes based on the ISRM. Compared with multiple clinical factors, the risk score of the ISRM was a significant risk factor and had a stronger ability to predict prognosis. The immune checkpoint molecules (CTLA4, PDCD1, TIGIT, and LAG3) were highly expressed in the low-risk group, suggesting that patients in the low-risk group had considerable improvement in immunotherapy [41,42]. Drug-sensitivity analysis showed that high-risk groups were more sensitive to Ulixertinib, BI-2536, ERK-1714, and Erlotinib. The ISRM also can predict ICI treatment outcomes of patients with lung cancer undergoing anti-PD-1 treatment [43]. Patients in the high-risk ISRM group had worse survival and lower expression of immune checkpoints, which are resistant to immunotherapy. In conclusion, the ISRM can predict survival status and better promote the understanding of anti-tumor therapy and immunotherapy for lung cancer.

Immunosenescence risk biomarkers have considerable power to predict survival outcomes of lung cancer and may be valuable tools to stratify patients to achieve personalized treatment of lung cancer. The ISRM offers a less invasive and perhaps more accurate protocol for lung cancer patients of different ages and with different immunity in clinical practice. Given the complexity of current clinical trials and the resources required, prognostic biomarkers could be a potential measure to accelerate clinical trials. ISRM biomarkers associated with clinical trial endpoints can identify patients who are more likely to experience the outcome, thereby improving the efficiency of clinical trials. The ISRM can be used for risk stratification of lung cancer-related mortality and response to immunotherapy, and shows good performance. If confirmed in future studies with a sufficiently long follow-up period, the powerful prognostic performance of the ISRM could become a cornerstone for risk stratification and personalized therapy in lung cancer patients.

## 5. Conclusions

We developed an immunosenescence gene set (ISGS) and examined the functional annotation ability of the ISGS across 33 tumor types and 29 normal tissues. Immunosenescence altered the heterogeneity of the tumor microenvironment, according to bulk and single-cell transcriptomic analysis. The immunosenescence risk model (ISRM) can precisely stratify the survival of lung cancer, with patients with a high-risk score having a worse survival and being resistant to immunotherapy. Our study contributed to the understanding of antitumor therapy and immunotherapy for lung cancer.

## Data Availability

All the expression and survival data of TCGA can be obtained from the UCSC Xena database (http://xena.ucsc.edu/public/, accessed on 10 November 2022). The gene expression matrix and related information of normal tissues were obtained from the GTEx database (https://www.gtexportal.org/, accessed on 22 November 2022). The expression and survival data for validation can be obtained from the Gene Expression Omnibus by accession numbers GSE40419, GSE209891, GSE68465, GSE72094, GSE26939, GSE68571, and GSE93157. The scRNA-seq in this study can be downloaded from the GSE144945 and DISCO (https://www.immunesinglecell.org/, accessed on 5 February 2023). The drug-sensitivity data of cell lines were obtained from the GDSC (http://www.cancerrxgene.org/, accessed on 13 May 2023). Immunohistochemical (IHC) staining data can be downloaded from HPA (https://www.proteinatlas.org, accessed on 27 July 2023). The analysis results associated with this paper are available in the figshare repository (https://figshare.com/s/665ec33ff0077e525ce0, uploaded on 11 June 2024). The bioinformatics analysis code has been uploaded to GitHub (https://github.com/Zhang-Yakun/ISRM_workflow, uploaded on 22 November 2023).

## Supplementary Materials

The following supporting information can be downloaded at: https://www.mdpi.com/article/10.3390/cancers17010085/s1, Figure S1: Overview of the genomic information of immunosenescence gene set (ISGS); Figure S2: Markers of annotated cell types in the GSE144945 datasets; Figure S3: Markers of annotated cell types in the GSE144945 datasets; Figure S4: Single-cell expression analysis and trajectory analysis of cell clusters which related with immunosenescence (GSE144945); Figure S5: Expression of up-regulated immunosenescence genes were significantly associated with immune factors; Figure S6: Cluster analysis and function analysis of PPI network in ISGS; Figure S7: The verification of ISRM in TCGA and GEO; Figure S8: Potential anti-cancer drug with prognostic risk; Figure S9: Small molecule drugs which were more sensitive in senescent LUAD cell lines; Table S1: Sample information of TCGA; Table S2: Sample information of GTEx; Table S3: Sample information of GEO cohort; Table S4: Description of scRNA-seq data in DISCO database; Table S5: Immune factors were downloaded from the TISIDB database; Table S6: Drug sensitivity data of cell lines obtained in the GDSC database; Table S7: A complete list of genes in immunosenescence gene set(ISGS); Table S8: Markers of cell clusters in DISCO database; Table S9: Motif rankings by iRegulon v1.3; Table S10: Summary of key up-regulated TF.

## Abbreviations

ISGS	Immunosenescence gene set
ISRM	Immunosenescence risk model
ICIs	Immune checkpoint inhibitors
TCGA	The Cancer Genome Atlas
GTEX	Genotype–tissue expression
LUAD	Lung adenocarcinoma
NK	Natural killer
TILs	Tumor-infiltrating lymphocytes
PPI	Protein–protein interaction
GSVA	Gene set variation analysis
ROC	Receiver operating characteristic
AUC	Area under the ROC curve
IC50	Half-maximal inhibitory concentration
XGBoost	eXtreme Gradient Boosting
