# Supplementary material for: Single-Cell and Bulk Transcriptomics Reveal the Immunosenescence Signature for Prognosis and Immunotherapy in Lung Cancer
Source: Cancers (Basel). 2024 Dec 30;17(1):85. doi: 10.3390/cancers17010085 (PMC11720133; doi:10.3390/cancers17010085)
Supplement: Supplementary file 1 [file cancers-17-00085-s001.zip › Supplementary Figures.pdf]

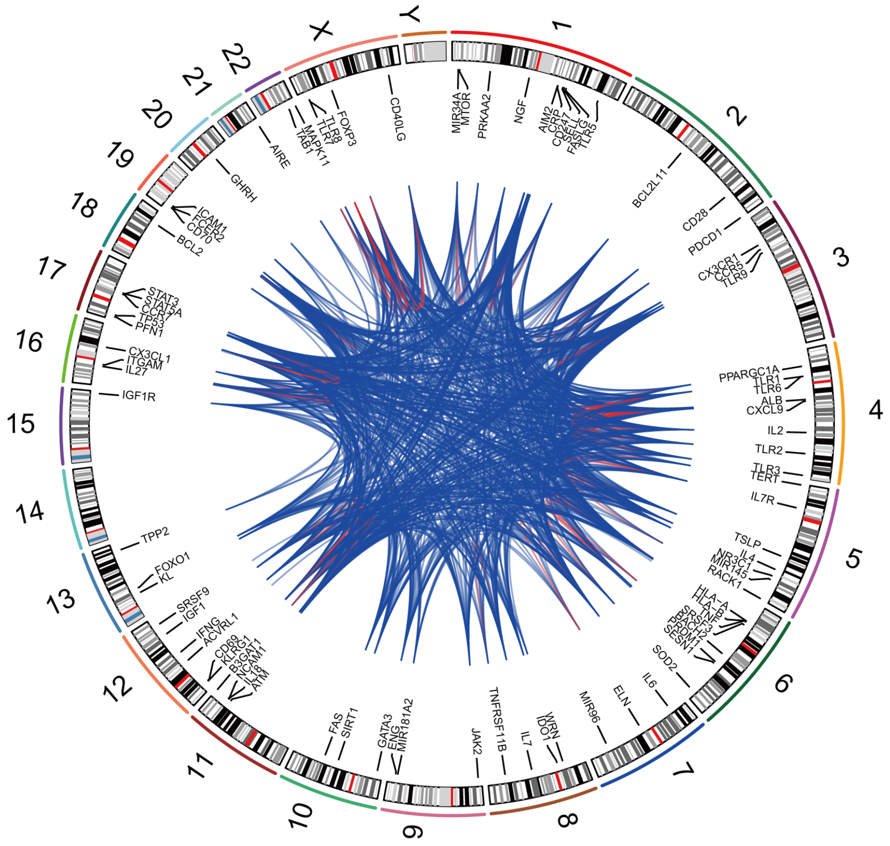


**Figure S1. Overview of the genomic information of immunosenescence gene set (ISGS).** Red lines will be used for junctions between the same chromosomal locations and blue lines for junctions between different chromosomes.

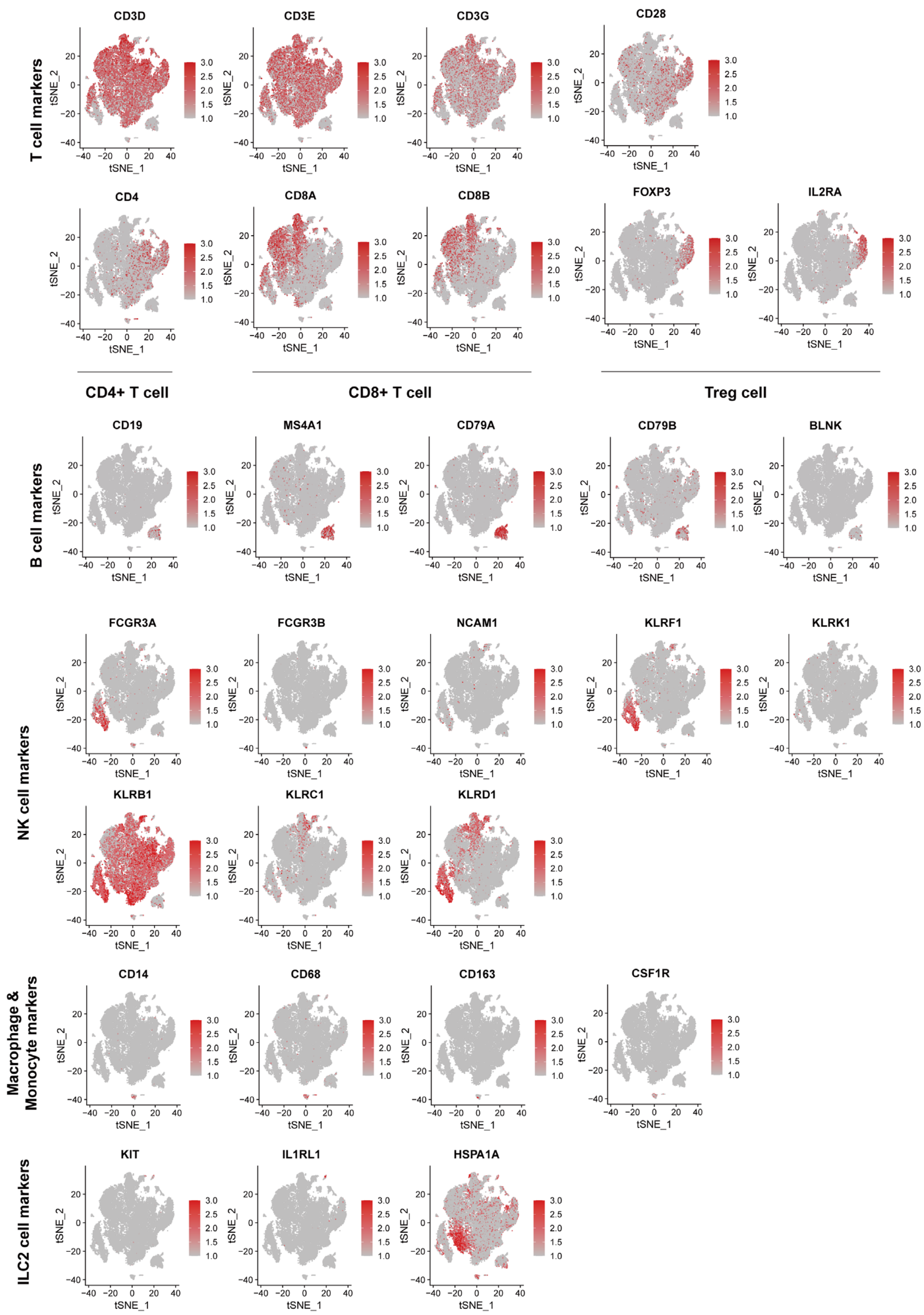


**Figure S2. Markers of annotated cell types in the GSE144945 datasets.** The scatter map showed the expression level of cell markers in single cells.

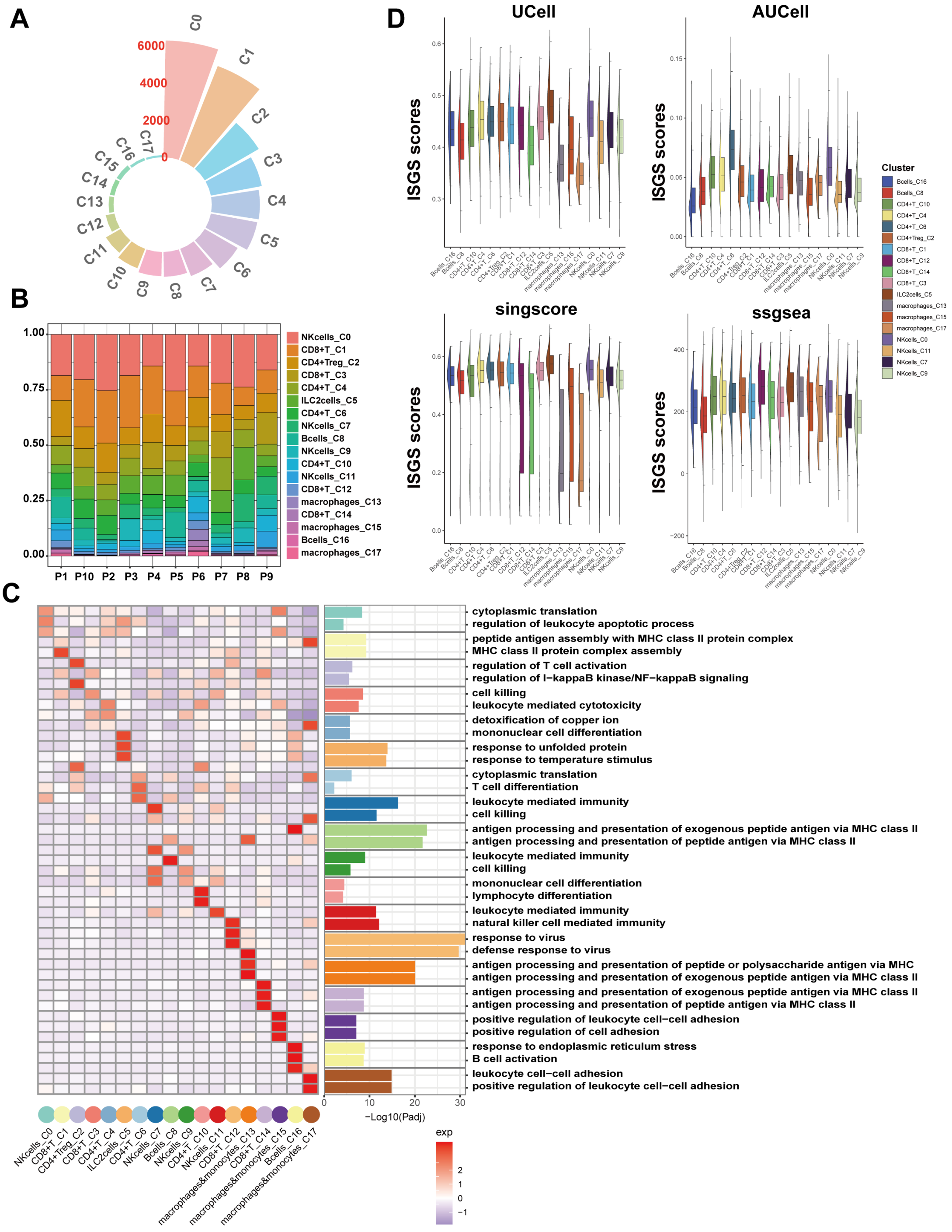


**Figure S3. The single-cell functional analysis of GSE144945. (A)** The bar plot showed the number of cells in clusters. **(B)** Percentage plots demonstrated the type and abundance of cells included in each patient. **(C)** The heat map showed the top 2 functional pathways enriched by markers of 17 cell types. **(D)** The ISGS scores of cell clusters calculated by the four methods (UCell, AUCell, singscore, ssgsea).

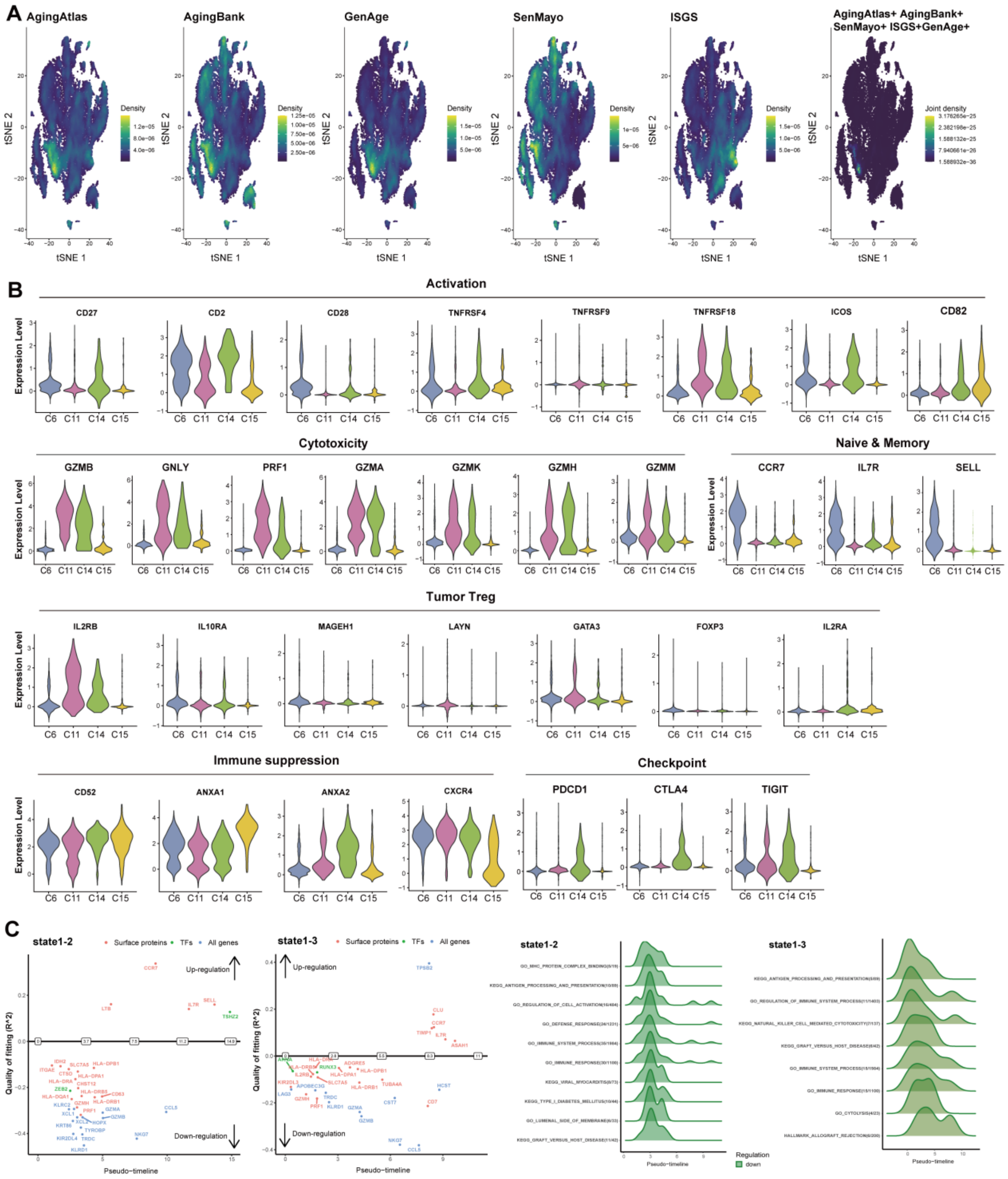


**Figure S4. Single-cell expression analysis and trajectory analysis of cell clusters which related with immunosenescence (GSE144945). (A)** The distribution of 5 senescence-related gene sets in single cells. **(B)** The expression levels of immune molecules in immunosenescence cell clusters.**(C)** The switch genes during differentiation of immunosenescence cell clusters by Geneswitch (left), and the pathways perturbed by state 1-2 and state 1-3, respectively (right).

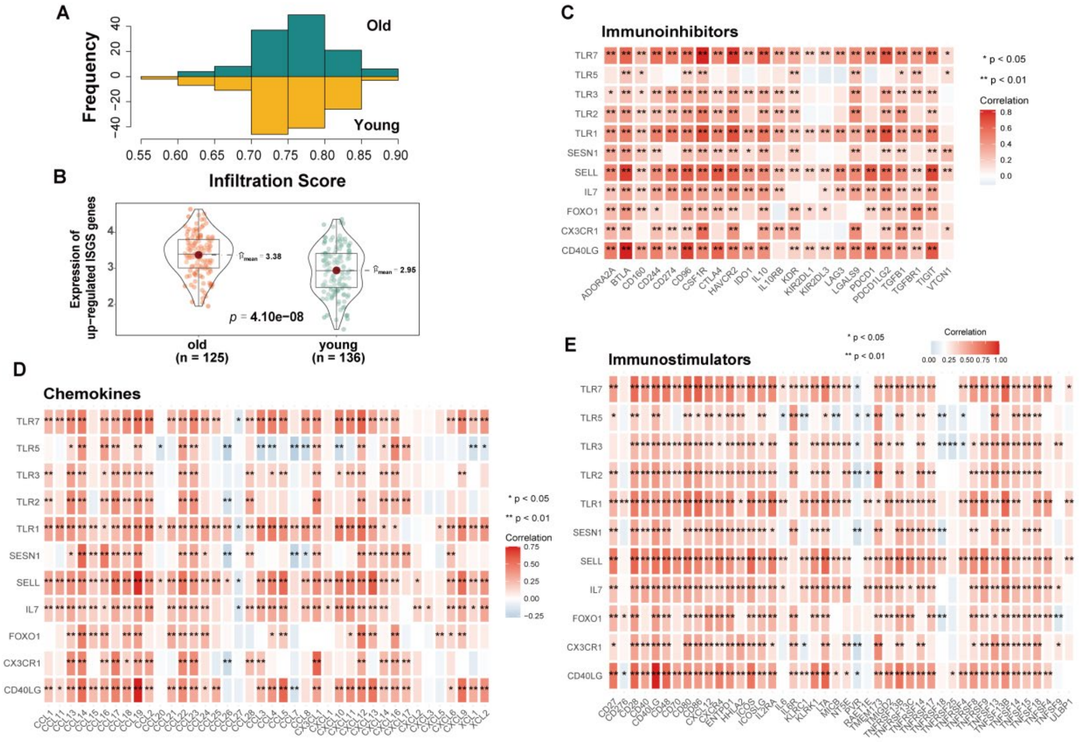


**Figure S5. Expression of up-regulated immunosenescence genes were significantly associated with immune factors.** **(A)** Distribution of immune infiltration scores between old and young groups. Green represents old group; yellow represents young group. **(B)** Boxplot of 11 significantly up-regulated ISGS genes (p=4.10e-08, t-test). The spearman correlation coefficients of up-regulated genes and immunoinhibitors **(C)**; chemokines **(D)**; immunostimulators **(E)**. *: p <= 0.05; **: p <= 0.01.

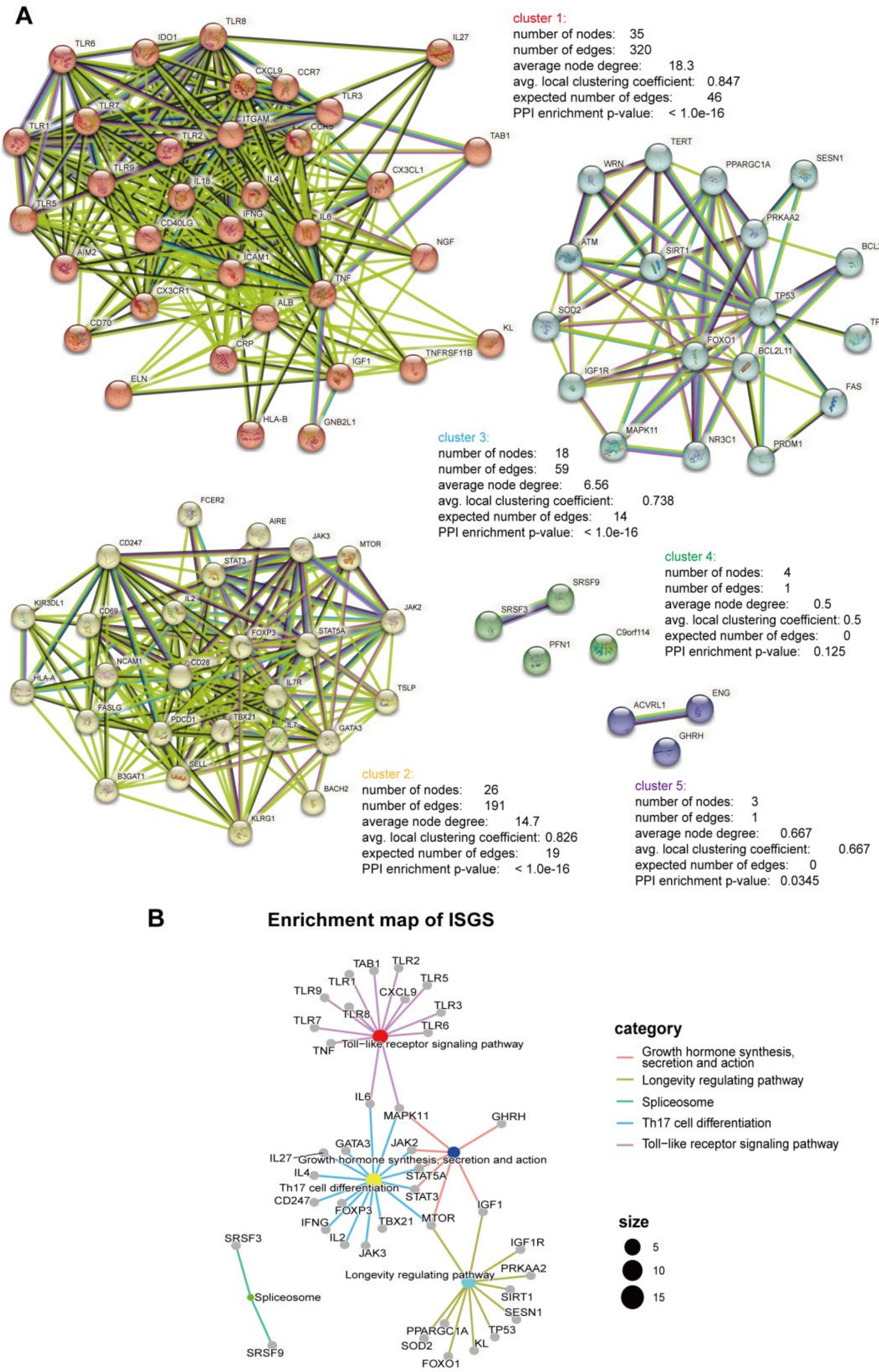


**Figure S6. Cluster analysis and function analysis of PPI network in ISGS. (A)** Five clusters of PPI network by k-means analysis. Nodes represent proteins, lines represent interactions. **(B)** Gene clusters included in the ISGS gene set were significantly enriched with different pathways. The circle size depicts number of pathways of clusters.

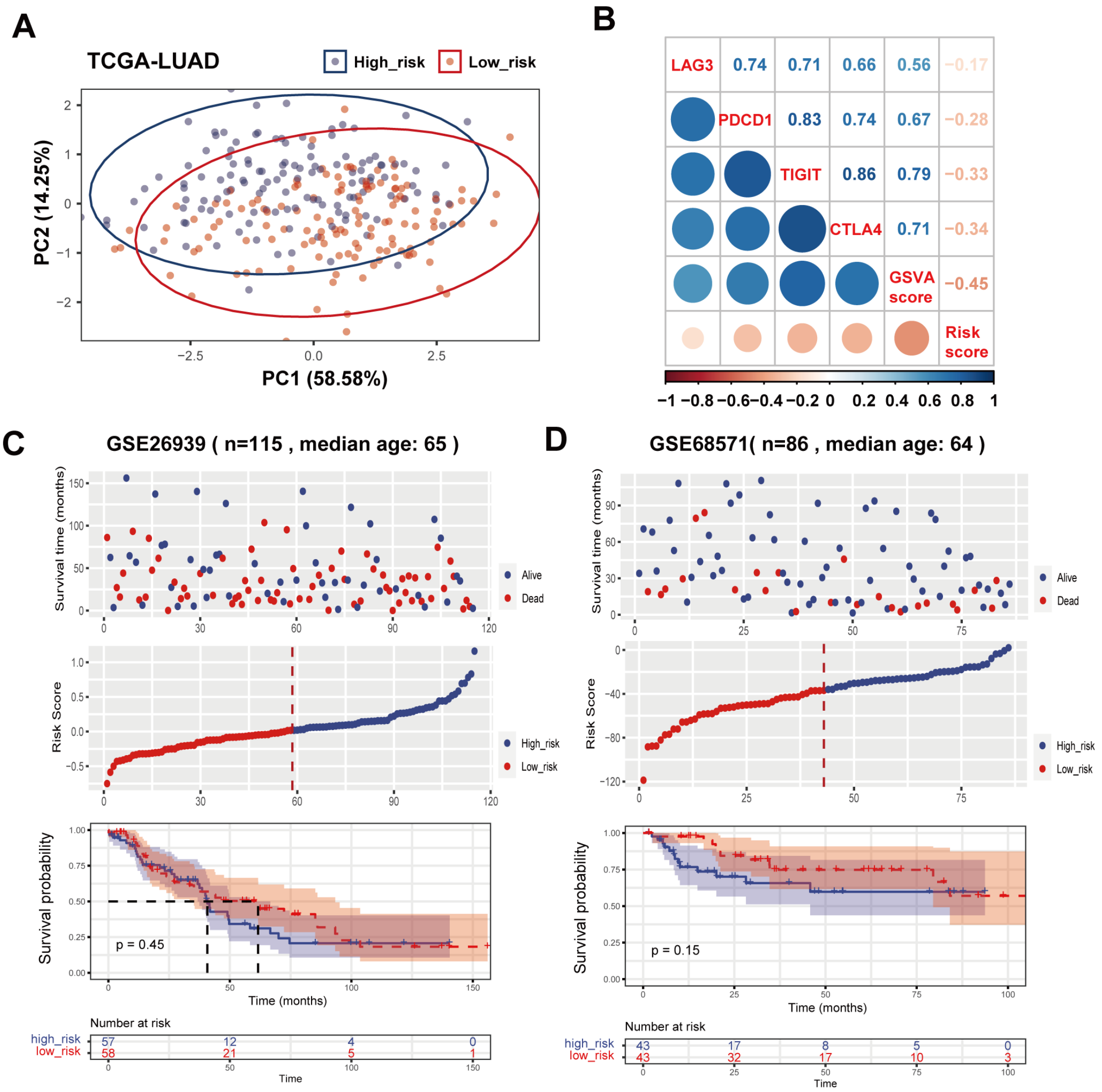


**Figure S7. The verification of ISRM in TCGA and GEO. (A)** PCA plot of TCGA LUAD cohort. Blue points represent high-risk group, and red points represent low-risk group. **(B)** Pearson correlation plot between immune checkpoint molecules and Risk score. The survival analysis of the GSE26939 cohort **(C)** and the GSE68571 cohort **(D)** performed by ISRM. LUAD, lung adenocarcinoma; PCA, principal component analysis.

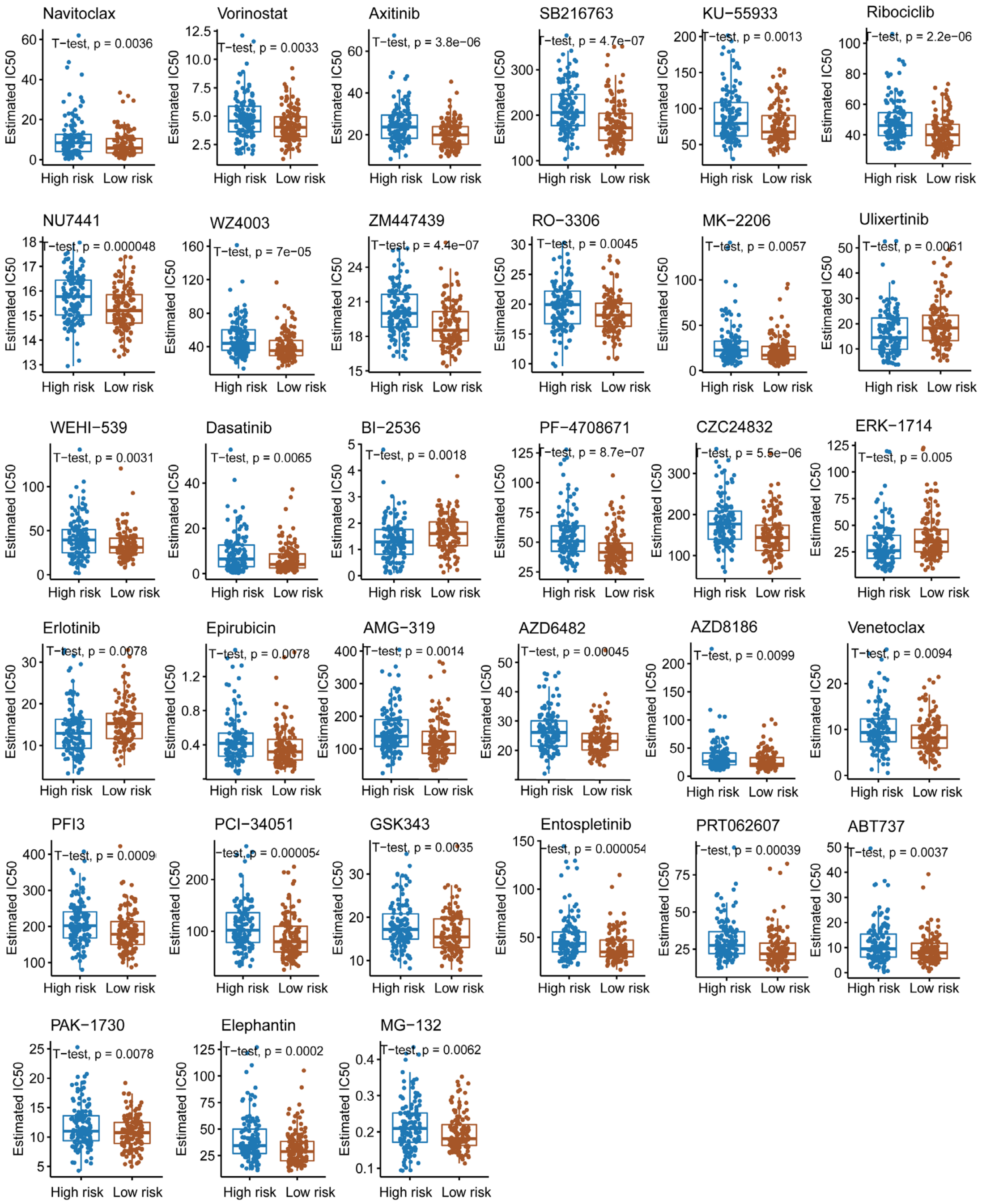


**Figure S8. Potential anti-cancer drug with prognostic risk.** Estimated IC50 of anti-cancer drugs grouped by Risk scores in TCGA LUAD cohort. All comparisons shown are statistically significant (T-test, $p<0.05$). Blue represents high-risk group; Orange represents low-risk group.

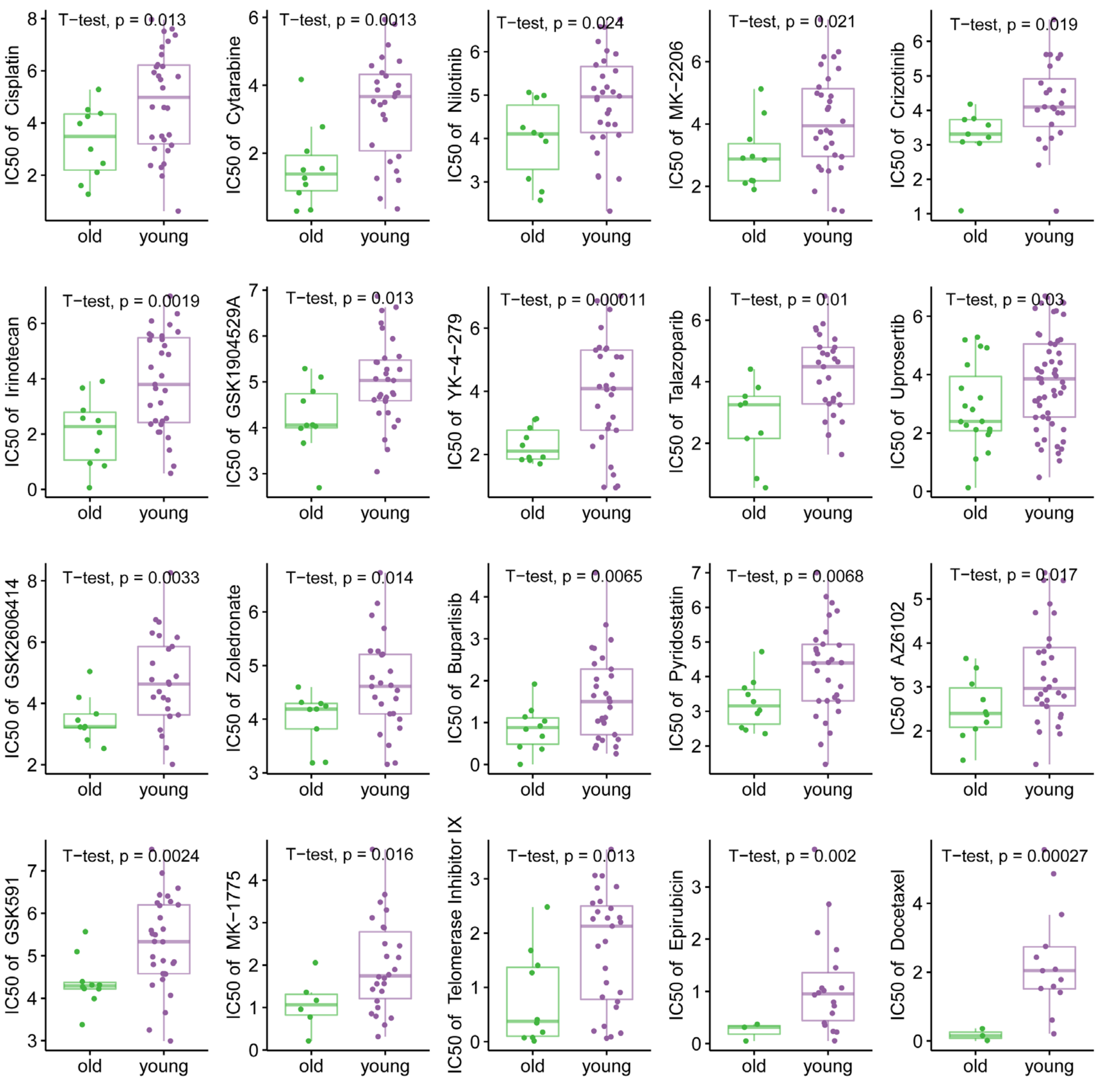


**Figure S9. Small molecule drugs which were more sensitive in senescent LUAD cell lines.** Drug response data for small molecule drugs were obtained from the GDSC database. All comparisons shown are statistically significant (T-test, $p<0.05$). Green represents old cell lines; Purple represents young cell lines.
